# Metabolic Engineering of *Escherichia coli* for Production of a Bioactive Metabolite of Bilirubin

**DOI:** 10.3390/ijms25179741

**Published:** 2024-09-09

**Authors:** Huaxin Chen, Peng Xiong, Ning Guo, Zhe Liu

**Affiliations:** School of Life Sciences and Medicine, Shandong University of Technology, Zibo 255049, China; xiongp@sdut.edu.cn (P.X.); guoning0312@163.com (N.G.); lzaaaa24@163.com (Z.L.)

**Keywords:** bilirubin, biliverdin, *Escherichia coli*, heme

## Abstract

Bilirubin (BR) is an important ingredient of a valuable Chinese medicine, Calculus bovis. Over recent decades, increasing evidence has confirmed that BR offers health benefits in cardiovascular health, stroke, diabetes, and metabolic syndrome. However, BR is mainly produced by extraction from pig bile. In this study, we assembled an efficient pathway for BR production by metabolic engineering of *Escherichia coli*. First, heme oxygenase (HO1) and biliverdin reductase were co-expressed in *E. coli*. HPLC and LC–MS confirmed the accumulation of BR in the recombinant *E. coli* cells. To improve BR production, the catalytic abilities of HO1 from different species were investigated. In addition, the outermembrane-bound heme receptor (ChuA) and the enzymes involved in heme biosynthesis were overexpressed among which ChuA, 5-aminolevulinic acid dehydratase (HemB), protoporphyrin oxidase (HemG), and ferrochelatase (HemH) were found to enhance BR accumulation in *E. coli*. In addition, expression of ferredoxin (Fd) was shown to contribute to efficient conversion of heme to BR in *E. coli*. To increase supply of NADPH, isocitrate dehydrogenase (IDH), NAD kinase (nadK), NADP-specific glutamate dehydrogenase (gdhA), and glucose-6-phosphate 1-dehydrogenase (ZWF) were overexpressed and were found to enhance BR accumulation when these proteins were expressed with a low-copy plasmid pACYCduet-1. Modular optimization of the committed genes led to a titer of 17.2 mg/L in strain M1BHG. Finally, fed-batch fermentation was performed for the strains M1BHG and M1, resulting in accumulation of 75.5 mg/L and 25.8 mg/L of BR, respectively. This is the first report on biosynthesis of BR through metabolic engineering in a heterologous host.

## 1. Introduction

**Figure 1:**
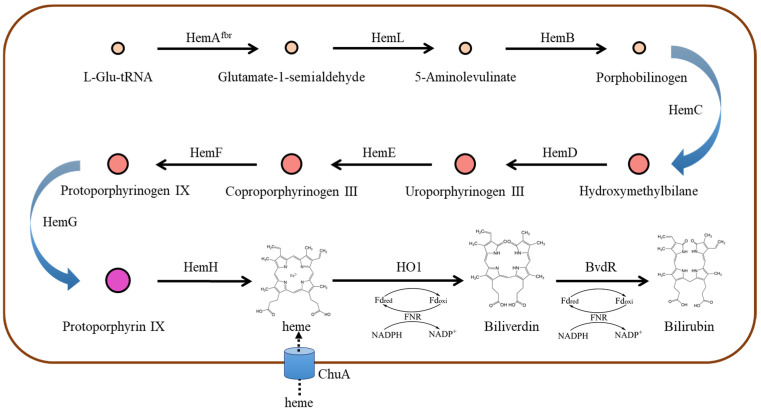
Schematic presentation of strategies for efficient BR biosynthesis in *E. coli*. HemA: glutamyl-tRNA reductase, HemL: glutamate-1-semialdehyde aminotransferase, HemB: 5-aminolevulinic acid (ALA) dehydratase, HemC: porphobilinogen deaminase, HemD: uroporphyrinogen III synthase, HemE: uroporphyrinogen decarboxylase, HemF: coproporphyrinogen III oxidase, HemG: protoporphyrin oxidase, HemH: ferrochelatase, ChuA: outermembrane-bound heme receptor.

Bilirubin (BR) is a yellowish open-chain tetrapyrrole pigment derived from heme catabolism. Heme is cleaved at the α-methene bridge ring by heme oxygenase (HO1), yielding an equimolar amount of biliverdin Ixα (BV), carbon monoxide, and iron [1]. BV is a water-soluble and relatively non-toxic compound. It can be reduced to less soluble and more toxic BR by the BV reductase (BvdR). Both reactions require NADPH as a reducing reagent (Figure 1) [2]. 

BR levels are tightly regulated in healthy adults, with concentrations ranging from 5 to 15 μM in the serum. However, BR levels in some patients change dramatically. Up to date, about 10% of children suffering from neonatal jaundice have high BR concentrations of up to 290 μM [1]. For a long time, BR was regarded as a waste product of heme catabolism. A high level of BR is often a sign of liver diseases such as clinical symptoms of kernicterus that often occur in infants [3]. Scientists tried to find ways to eliminate the harmful waste product from patients using phototherapy. Later, researchers found that a mildly elevated BR level protects against diseases related to elevated oxidative stress, an exaggerated immune response, and metabolic dysfunction [1]. Over the past several decades, increasing studies have reported the beneficial associations of mildly elevated systemic BR levels with diseases of civilization [4,5,6]. BR offers health benefits in cardiovascular health, stroke, diabetes, and metabolic syndrome [1,3]. Therefore, manipulation of serum BR levels is considered a potential strategy to cope with a range of health issues.

BR is a main component of Calculus bovis, a famous traditional animal drug in China and Japan that has been widely used for thousands of years to treat various diseases such as epilepsy, stroke, pediatric shock, and sore throat [7]. There is a high demand for Calculus bovis for traditional Chinese medicine. According to the NMPA, about 800 pharmaceutical companies produce medicines in which Calculus bovis is the main component (https://www.nmpa.gov.cn, (accessed on 10 May 2024)). Natural C. bovis is rare, expensive, and variable in quality. Calculus bovis artifactus, a mixture of cholic acid, hyodeoxycholic acid, taurine, BR, and other components, serves as an alternative [7]. Among these components, cholic acids and BR are considered the most important constituents. In Calculus bovis artifactus, however, BR concentration was reported to be 0.63%, which is much lower than that in natural Calculus bovis [8]. Increasing the BR content is required to improve the quality and efficacy of Calculus bovis artifactus.

Demand for BR is more than 50 tons per year [9]. Currently, BR is mainly extracted from animals such as pig and bovis [10]. Natural BR, however, brings potential risks due to infectious viruses such as mad cow disease and foot-and-mouth diseases. Furthermore, the extract is a mixture of different isomers, making BR purification complex. It is urgent to develop new strategies for efficient BR preparation. A Chinese patent declared a method that BR can be transformed by using heme as substrate and HO1 and BvdR as catalyst [11]. Recently, it was reported that BvdR was successfully expressed in recombinant *Escherichia coli* cells, which were employed as biocatalysts to transform BV to BR [12]. Although a conversion yield of 72.3% was obtained, this method is not applicable for industrial-scale preparation since BV is not available in bulk at present. A chemical synthesis method for BR production was reported that a number of 11 steps from butyrolactone to BR resulted in 19% overall yield with 98.9% purity [9]. This new synthetic pathway makes it possible to produce BR on an industrial scale.

In this study, we adopt metabolic engineering for the production of BR by recombinant *E. coli*. First, HO1 and BvdR were co-expressed in *E. coli* to examine whether BR could be produced in *E. coli*. After identification of BR by HPLC and LC–MS, HO1 with high activity/expression level was screened from several species. Next, heme supplementation to culture media and expression of a heme transporter (ChuA) were carried out in an attempt to enhance heme absorption. In addition, the effects of overexpression of heme biosynthesis pathway genes, ferredoxin (Fd), and NADPH regenerating genes on BR production were examined. Finally, modular optimization of the committed genes was performed to further increase the titer of BR production. BR titer in the final strain M1BHG reached 75.5 mg/L in a 5 L bioreactor.

## 2. Results and Discussion

### 2.1. Construction of Pathway for BR Biosynthesis in E. coli

**Figure 2:**
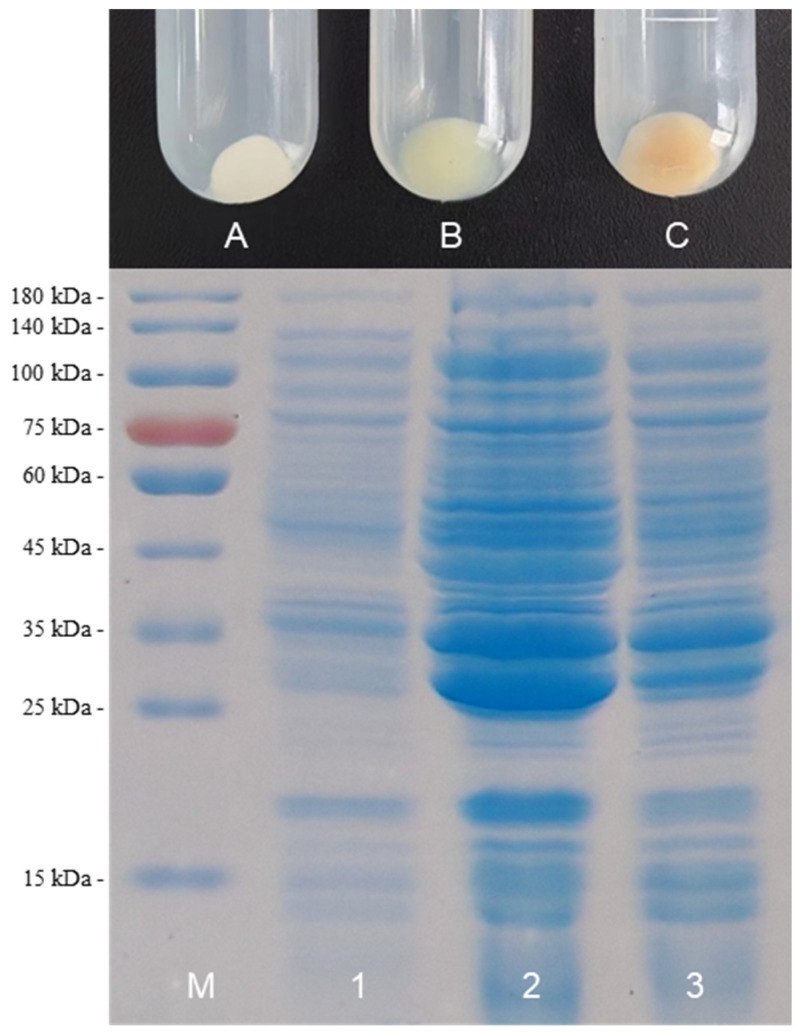
Harvested pellets of the *E. coli* M2 cells (top) and SDS-PAGE of the proteins extracted from the *E. coli* M2 cells (bottom). A: non-induced cell pellets; B: cell pellets after IPTG induction for 24 h; C: cell pellets after IPTG induction for 48 h. M: protein marker; 1: proteins extracted from non-induced cell pellets; 2: proteins extracted from cell pellets after IPTG induction for 24 h; 3: proteins extracted from cell pellets after IPTG induction for 48 h.

To construct a pathway for BR biosynthesis, *HO1* and *bvdR* from *Synechocystis* sp. PCC6803 were codon-optimized and expressed in *E. coli* BL21(DE3). The recombinant *E. coli* M2 cells were induced at 30 °C to promote soluble expression of foreign proteins. The *E. coli* cells turned pale green after induction by IPTG for 24 h and turned pale orange after induction by IPTG for 48 h (Figure 2). SDS-PAGE showed that compared with the non-induced cells, there were two obvious bands that matched the theoretical molecular weight of 6HO1 (26.9 KDa) and BvdR (36.6 kDa), respectively, for the IPTG-induced M2 cells (Figure 2). These results indicated that HO1 and BvdR have been successfully expressed in the recombinant *E. coli* cells and might transform heme into BR. The product was then extracted by methanol and identified by HPLC and LC–MS (Figure S2). HPLC analysis of the cell extracts showed that there was an elution peak around 14.5 min, identical to that for the standard BR. LC–MS analysis showed that m/z was 585.27, which is consistent with the known molecular weight of BR (C_33_H_36_N_4_O_6_, molecular weight: 584.66). These results showed that the pathway for BR was successfully constructed, and BR had accumulated in the recombinant *E. coli* cells.

### 2.2. Screening of HO1 to Improve BR Production

**Figure 3:**
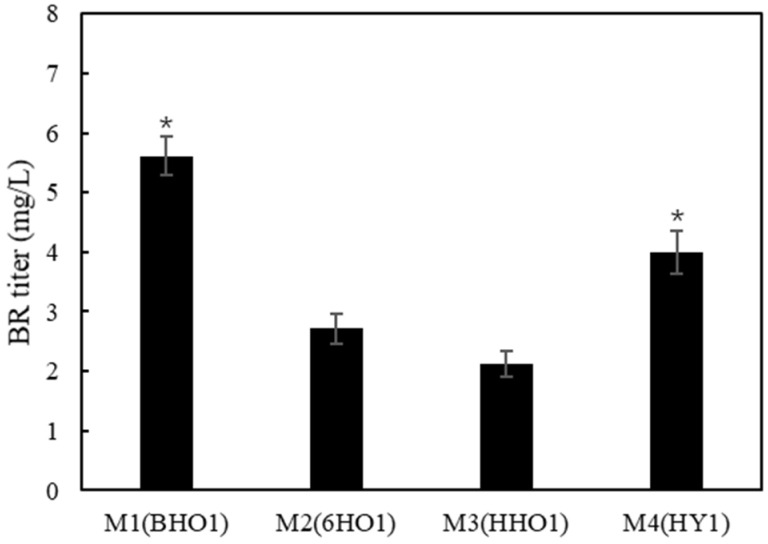
Screening of HO1 derived from different species based on BR production in recombinant *E. coli*. BHO1: HO1 from *T. elongatus* BP-1; 6HO1: HO1 from *Synechocystis* sp. 6803; HHO1, HO1 from *Homo sapiens*; YHO1: HY1 from *Arabidopsis thaliana*. Asterisks show the significant difference (*p* < 0.05) compared with the control (strain M2).

HPLC analysis showed that BV was not detected in the *E. coli* M2 cells (Figure S1), suggesting that the HO1-catalyzed conversion of heme to BV is a rate-limiting step during BR production. It is reasonable that increasing the activity and/or expression level of HO1 could improve BR production. Several HO1s from different species were evaluated. The *HO1* gene from *Thermosynechococcus elongatus* BP-1 (BHo1), human (HHo1), and *Arabidopsis thaliana* (HY2) was codon-optimized and co-expressed with *bvdR* in *E. coli*. Compared with the strain M2, the strain M1 had the highest level in BR production (5.6 mg/L, Figure 3). Our previous study showed that among HO1s from several algal species, the recombinant HO1 from *T. elongatus* BP-1 had the best performance for phycoerythrobilin (PEB) and phycocyanobilin (PCB) production. In another study, however, it was reported that HO1 from *Synechocystis* sp. PCC 6803 was superior to BHO1 for PCB synthesis in *E. coli* [13]. This discrepancy could be due to the fact that in our previous study the *BHO1* gene was optimized for *E. coli*, and thus the higher expression level improved BV production. Therefore, the codon-optimized *BHO1* gene was used for the following study.

### 2.3. Effects of Heme Supplementation on BR Accumulation

**Figure 4:**
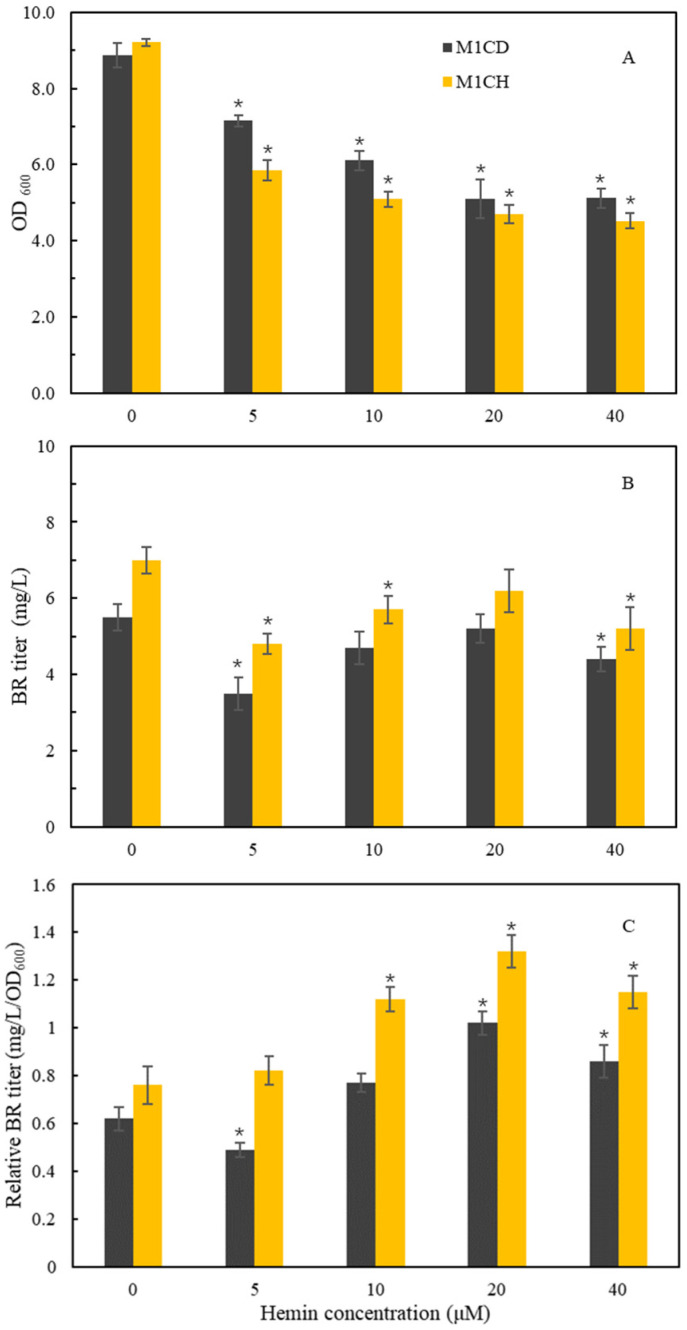
Effects of hemin supplementation on cell growth (**A**), BR titer (**B**), and relative BR titer (**C**), which was assessed by BR titer divided by cell density in the strain M1CD (black) and the strain M1CH (orange). Asterisks show the significant difference (*p* < 0.05) compared with the control.

Heme is the substrate for BR production. Biosynthesis of heme involves a number of genes and is tightly regulated to maintain a low level of heme (Figure 1) [14]. A simple approach seems to be the supplementation of heme to culture media. In the present study, hemin was added to the TB medium at the start of the induction phase. For the stain M1, decreasing BR titer and cell growth were observed with increasing concentrations of hemin (Figure 4A,B). When relative BR titer was assessed by BR titer divided by OD_600_, an increased relative BR titer was observed with increasing concentrations of hemin (Figure 4C). These results indicated that hemin is toxic to cell growth and led to the decreased BR production. Nevertheless, the results also indicated that hemin supplementation could improve BR production on a biomass basis. Generally, *E. coli* strains have limited ability to use exogenously added heme. Previous studies showed that co-expression of ChuA, an outermembrane-bound heme receptor from *E. coli* O157:H7, increased the heme content and activity of catalase in *E. coli* [15,16]. To promote uptake of heme, ChuA is co-expressed with HO1 and BvdR in the recombinant *E. coli* cells, generating the strain M1CH. Similar effects of hemin supplementation on cell growth, BR titer, and relative BR titer were observed in the strain M1CH (Figure 4). Notably, whether or not hemin is added to the culture, there were higher BR titers and relative BR titers in the strain M1CH than in the control strain M1CD (Figure 4). The results suggested that ChuA offers the recombinant *E. coli* cells the ability to withdraw heme from the extracellular environment. More importantly, the findings indicated that increasing heme availability might be a feasible approach to improving BR production.

### 2.4. Regulation of Heme Biosynthesis and Its Effects on BR Biosynthesis

**Figure 5:**
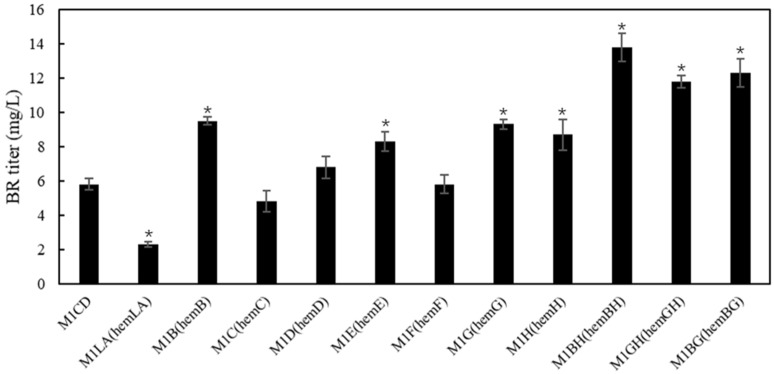
Effects of co-expressing heme biosynthesis pathway genes on BR production in recombinant *E. coli* cells. The recombinant *E. coli* strains co-expressed HO1 and bvdR with *hemLA*, *hemB*, *hemC*, *hemD*, *hemE*, *hemF*, *hemG*, *hemH*, *hemBH*, *hemGH*, *hemBG*, respectively. Strain M1CD was the control. Asterisks show the significant difference (*p* < 0.05) compared with the control (strain M1CD).

Heme biosynthesis in *E. coli* involves a number of genes, including *hemA*, *hemL*, *hemB*, *hemC*, *hemD*, *hemE*, *hemF*, *hemG*, and *hemH* (Figure 1). Improvement in heme biosynthesis could be achieved by up-regulation or down-regulation of key genes [17], offering an economic strategy to enhance BR production since no additional substrate is needed to add into the culture. It has been accepted that ALA biosynthesis is the rate-limiting step for the heme biosynthesis [18]. Overexpression of *hemL* and *hemA* could burst heme accumulation [19]. In this study, *hemL* (from *E. coli*) and *hemA^S^* (a mutant of *hemA* from *Salmonella Arizona* [20]) were designed as a chimeric gene (*hemLA*). The *hemLA* gene was co-expressed with HO1 and BvdR (strain M1LA). However, co-expression of *HemLA* dramatically reduced the BR production. In our previous studies, we noticed that co-expression of *HemLA* or supplementation of ALA in culture media reduced BV, PEB, and PCB production in recombinant *E. coli* cells (unpublished data). In these recombinant cells, HO1 was the common enzyme responsible for the conversion of heme to BV. A previous study reported that overexpression of *hemB* decreased ALA level in recombinant *E. coli* cells [21]. Interestingly, we found that BR titer was improved in *hemB*-overexpressing *E. coli* cells (Figure 5). Collectively, we hypothesize that ALA is one of the important factors regulating HO1 activity. That is, a higher level of ALA inhibits HO1 activity and reduces the production of BV and thus the final compounds (BR, PEB, or PCB), while a low level of ALA causes opposite effects.

To investigate the effects of overexpression of other heme genes on BR biosynthesis, *hemC*, *hemD*, *hemE*, *hemF*, *hemG*, and *hemH* were individually co-expressed with HO1 and BvdR by using the plasmid pCDFduet-1. The results showed that there were distinct effects on BR accumulation (Figure 5). Among these genes, overexpression of *hemB*, *hemE*, *hemG*, or *hemH* improved BR accumulation, while *HemC* inhibited BR biosynthesis. A previous study reported that over-expression of *hemB*, *hemG*, or *hemH* genes reduced the production of ALA, while down-regulation of *hemB* or *hemH* decreased the heme biosynthesis from ALA [21]. Another study also reported that overexpression of the *hemG* led to higher accumulation of heme [22]. All these findings suggested that the enhanced BR production in *hemB*, *hemG*, or *hemH*-overexpressing *E. coli* cells could be attributed to an increased metabolic flux from ALA to heme.

In an attempt to further enhance BR biosynthesis, *hemB*, *hemG*, and *hemH* were co-expressed with combinations of *hemB* and *hemH* (strain M1BH), *hemB* and *hemG* (strain M1BG), and *hemG* and *hemH* (strain M1GH). Figure 5 showed that the strain M1BH produced the highest level of BR (13.8 mg/L), followed by the strain M1BG (12.3 mg/L) and strain M1GH (11.8 mg/L). It is reported that accumulation of intermediate protoporphyrinogen IX would cause feedback inhibition of HemB [22]. Overexpression of *hemG* or *hemH* facilitates the conversion of protoporphyrinogen IX to protoporphyrin IX or heme, increasing the metabolic flux from ALA to heme and BR [18,22]. It is likely that co-expression of *hemB*, *hemG*, and *hemH* together would further enhance BR biosynthesis.

### 2.5. Regulation of BR Production by Ferredoxin (Fd) and Ferredoxin-NADP^+^ Reductase (FNR)

**Figure 6:**
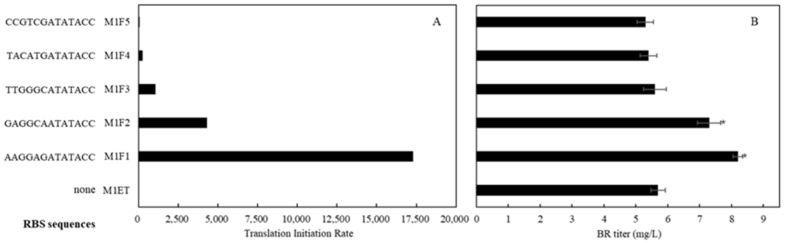
Fine turning of Fd expression levels by ribosomal binding site (RBS) design and their effects on BR production. (**A**) Translation initiation rates for the designed RBSs. (**B**) BR production by recombinant strains in which transcription of Fd was initiated by the designed RBSs. Asterisks in the figure indicate the difference (*p* < 0.05) compared with the control strain M1ET.

Most cells have endogenous Fd and FNR activities. However, several recent studies have shown that in some cells Fd activity is a limiting factor during the conversion of heme to PCB, which is catalyzed by HO1 and 3Z-phycocyanobilin:ferredoxin oxidoreductase (PcyA) [23,24]. In this study, BR can be detected in recombinant *E. coli* cells, suggesting that Fd activity has existed in *E. coli* cells (Figure 6). However, it is unknown whether Fd is sufficient for the conversion of heme into BR in the recombinant *E. coli* cells. Therefore, Fd and FNR were co-expressed by plasmid pETDuet-1, with the Fd gene ligated into the first expression cassette and the FNR gene into the second expression cassette. To fine tune the expression level of Fd, the RBS sites were designed to control translation initiation rate with a factor of 4 over a range of 256-fold (Figure 6) [25]. Five expression plasmids and strains were constructed. Strain M1FF1 harbored the original RBS of pETDuet-1 to drive Fd expression. Compared with the control strain M1ET, the strains M1FF1 and M1FF2 produced higher levels of BR, while in other strains, expression of Fd-FNR did not affect BR accumulation. These results indicated that additional Fd activity contributes to the conversion of heme to BR in the recombinant *E. coli* cells.

### 2.6. Overexpression of ZWF, nadK, gdhA and IDH for Improving NADPH Supply on BR Biosynthesis

**Figure 7:**
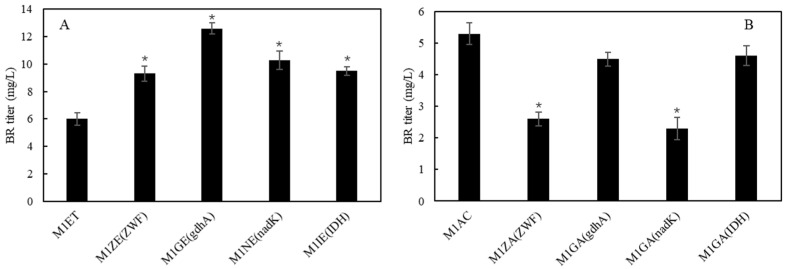
Effects of individual co-expression of NADPH generating genes *NAD*, *ZWF*, *gdhA* and *IDH* on BR production. (**A**) *NAD*, *ZWF*, *gdhA*, *IDH* genes were individually co-expressed by using a low-copy plasmid pACYCduet-1. (**B**) *NAD*, *ZWF*, *gdhA*, *IDH* genes were individually co-expressed by using a medium-copy plasmid pETduet-1. Asterisks show the significant difference (*p* < 0.05) compared with the control (strain M1ET or M1AC).

Reduced nicotinamide adenine dinucleotide phosphate (NADPH) is an essential electron donor in bacteria and other organisms. It is the driving force for most biosynthetic enzymatic reactions. In the process of BR biosynthesis, NADPH acts as a cofactor for the formation of BV and BR. Increasing intracellular NADPH availability is a strategy to improve accumulation of NADPH-dependent products [26,27]. It has been shown that introduction of the Entner–Doudoroff pathway from *Zymomonas mobilis* into *E. coli* MG1655 increased the NADPH regeneration rate 25-fold and thus improved terpenoid production [28]. In the present study, isocitrate dehydrogenase (IDH), NAD kinase (nadK), NADP-specific glutamate dehydrogenase (gdhA), and glucose-6-phosphate 1-dehydrogenase (ZWF) were individually co-expressed with pACYCDuet-1 in *E. coli*. Compared to the control strain (M1AC), the strains overexpressing NADPH regenerating genes significantly enhanced BR biosynthesis. Among these strains, the strain overexpressing the *gdhA* gene (strain M1GE) accumulated the highest level of BR, reaching a titer of 12.6 mg/L (Figure 7). The results indicated that the activities of HO1 and/or BvdR are limited by NADPH supply in the recombinant *E. coli* cells. To evaluate whether higher-level overexpression of these genes would further improve BR biosynthesis, these NADPH-regenerating genes were individually overexpressed by using the medium-copy plasmid pETDuet-1. However, no improvement of BR accumulation was observed in the recombinant *E. coli* cells. Instead, overexpression of these NADPH-regenerating genes led to a decrease in BR accumulation (Figure 7). It is speculated that excess expression of these genes led to negative effects on transcription and/or translation of HO1 and BvdR and therefore reduced BR production in the recombinant *E. coli* cells.

### 2.7. Modular Optimization of the Committed Genes for Further Improvement of BR Biosynthesis

**Figure 8:**
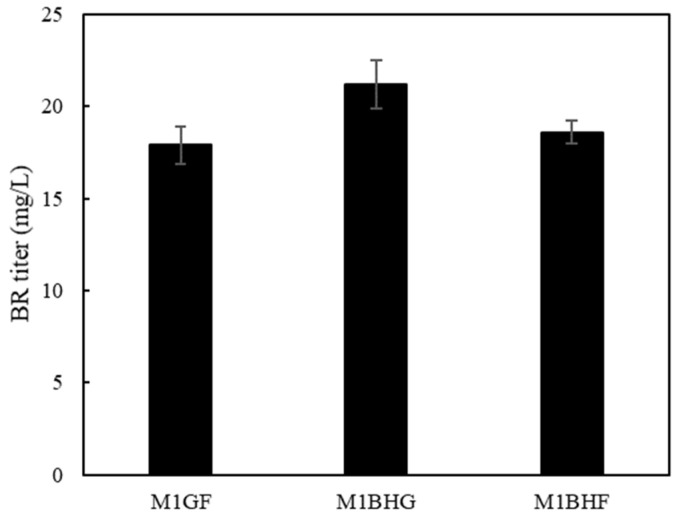
Modular optimization of BR production by combinational expression of *hemLA*, *Fd-FNR*, and *gdhA*.

Based on the above analysis, it could be expected that a combinational up-regulation of *hemBH*, *Fd/FNR*, and *gdhA* would further enhance BR production. To this end, these committed genes were overexpressed in recombinant *E. coli* cells. As shown in Figure 8, the strain M1BHG overexpressing *hemBH* and *gdhA* produced 17.2 mg/L of BR, while strain M1BHF overexpressing *hemBH* and *Fd/FNR* produced 14.6 mg/L of BR, and strain M1GF overexpressing *Fd/FNR* and *gdhA* produced 15.9 mg/L of BR. The results indicated synergistic effects of these committed genes on BR biosynthesis. The highest level of BR observed in the strain M1BHG suggested that a sufficient supply of heme and NADPH is more critical for the biosynthesis of BR in recombinant *E. coli* cells.

### 2.8. Fed-Batch Fermentation in a 5 L Bioreactor

**Figure 9:**
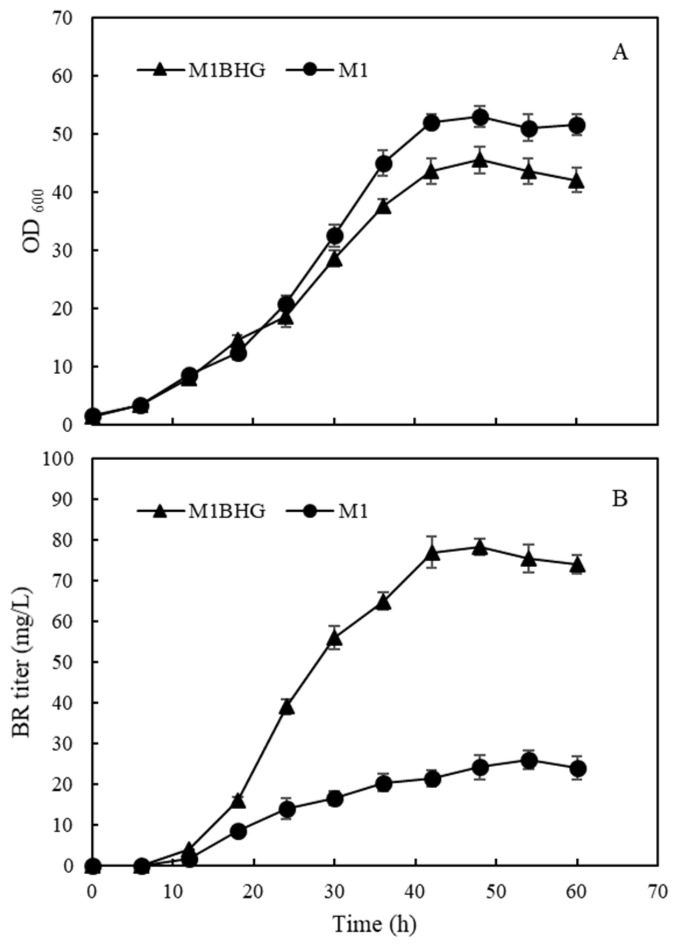
Time courses of cell growth (**A**) and BR titers (**B**) for the recombinant *E. coli* strain M1 and M1BHG in fed-batch fermentation.

During a large-scale cultivation, plasmid loss in recombinant *E. coli* cells generally leads to decreased protein expression or an incomplete pathway for the desirable product [29,30]. The strain M1BHG with the highest titer of BR among the strains in this study harbors three types of plasmids for co-expression of five genes. To investigate whether this strain exhibits superior performance over the starting strain M1, which harbors only one type of plasmid, both strains were cultivated in a 5 L bioreactor. Constant feeding mode was employed to achieve high cell density. Compared with the strain M1, strain M1BHG had a lower growth rate and lower cell density during the stationary phase (Figure 9). One possible explanation is that the expression of three additional genes (*hemB*, *hemH*, and *gdhA*) caused a heavy metabolic burden to *E. coli* cells. Alternatively, the reduced cell growth may be a result of an increased glutamate pool in the M1BHG *E. coli* cells. Overexpression of *gdhA* leads to an increase in the glutamate pool in *E. coli* cells, which is a precursor for the synthesis of many other metabolites and causes rebalance in cellular homeostasis [31]. Indeed, it has been reported that artificial addition of glutamate reduced ALA accumulation and cell growth in *E. coli* [21]. Both strains started to accumulate BR at 12 h, and then the BR titers increased with increasing cultivation time. M1BHG produced the highest titer of 78.3 mg/L BR at 48 h while M1 produced the highest titer of 25.8 mg/L BR at 54 h. These data suggested that although the strain M1BHG was suffered from plasmid loss, this strain was more efficient for BR biosynthesis compared with the strain M1. In our previous study, we found that the biosynthetic pathway constructed by using one expression plasmid is superior for recombinant phycobiliprotein production to that constructed by using multiple plasmids [32]. It is likely to further improve BR production by designing these foreign genes as ploycistron(s) and expressed with a single plasmid. This strategy is of importance since only one antibiotic is required to maintain plasmid stability, which confers a cost advantage, especially for a large-scale cultivation.

## 3. Conclusions

In conclusion, we have reported production of BR by metabolically engineered *E. coli*. Among several HO1, HO1 from *T. elongates BP-1* exhibited the best performance for BR production. Hemin supplementation and ChuA overexpression were shown to enhance BR production in *E. coli* on a biomass basis. In addition, up-regulation of HemB, HemG, or HemH was found to enhance BR production, demonstrating an important role of heme availability in BR biosynthesis. We also showed that sufficient Fd activity and NADPH regeneration were required for enhancing production of BR. Finally, modular optimization of the committed proteins led to the strain M1BHG with the highest titer of BR producing 78.3 mg/L BR in a 5 L bioreactor. To the best of our knowledge, this is the first report on the biosynthesis of BR in an engineered microorganism and will lay the foundations for the construction of microbial strains for industrial BR production in the future.

## 4. Materials and Methods

### 4.1. Chemicals

Chemicals. Artificial genes were synthesized by General Biologics (Anhui) Co., Ltd. (Chuzhou, China). Taq DNA Polymerase, Phanta Max Master Mix, and ClonExpress^®^ II were obtained from Nanjing Vazyme Biotechnology Co., Ltd. (Nanjing, China). All restriction enzymes were obtained from New England BioLabs (New England Biolabs, Ipswich, MA, USA). Bilirubin standard was obtained from Sigma-Aldrich (St. Louis, MO, USA). Other chemicals were purchased from Sangon Biotech (Shanghai) Co., Ltd. (Shanghai, China).

### 4.2. Plasmids and Strains

Table S1 lists all strains and plasmids constructed in the study. An *E. coli* strain for BR biosynthesis was constructed by co-expression of HO1 (denoted as 6HO1 hereafter) and BvdR from *Synechocystis* sp. PCC 6803 in *E. coli* strain BL21(DE3). This engineered strain (denoted as M2) contained plasmid pRSFduet-6HO1-BvdR with *6HO1* in the first expression cassette and *bvdR* in the second cassette. *HO1* genes from *T. elongatus* BP-1 (denoted as *BHo1*), human (denoted as *HHo1*), and *Arabidopsis thaliana* (*HY1*) were optimized according to *E. coli* codon usage and artificially synthesized. The *HO1* genes were digested with restriction enzymes and cloned into similarly digested pRSFduet-6HO1-BvdR, generating the plasmids pRSF-BHo1-BvdR, pRSF-HHo1-BvdR, and pRSF-HY2-BvdR, respectively. These plasmids were individually transformed into competent *E. coli* BL21(DE3) cells, generating strains BM1, HM1, and YM1, respectively. Heme biosynthesis pathway genes, including *hemB*, *hemC*, *hemD*, *hemE*, *hemF*, *hemG*, and *hemH*, were amplified from *E. coli* genomic DNA, gel purified and digested with BamHI, and ligated into similarly digested pCDFduet-1, respectively. The resulting plasmids pCDF-hemB, pCDF-hemC, pCDF-hemD, pCDF-hemE, pCDF-hemF, pCDF-hemG, and pCDF-hemH were individually co-transformed with the plasmid pRSF-BHo1-BvdR into competent *E. coli* BL21(DE3) cells, generating strains M1B, M1C, M1D, M1E, M1F, M1G, M1H, respectively. The *hemH* and *hemG* PCR products were digested with NdeI and ligated individually into similarly digested pCDF-hemB, yielding the plasmids pCDF-hemBH and pCDF-hemBG, respectively. The *hemG* gene was digested with NdeI and ligated into similarly digested pCDF-hemH, yielding plasmid pCDF-hemGH. The plasmids pCDF-hemBG, pCDF-hemBH, and pCDF-hemGH were individually co-transformed with pRSF-BHo1-BvdR, generating strains M1BG, M1BH, and M1GH, respectively. The *ChuA* gene was artificially synthesized by General Biology Company and cloned into plasmid pCDFduet-1, yielding pCDF-chuA. The plasmid pCDF-chuA or pCDFduet-1 were individually co-transformed with pRSF-BHo1-BvdR into *E. coli* cells, generating strains M1CH and M1CD, respectively. *hemL* and *hemA* were designed as a chimeric gene and ligated into the plasmid pCDFduet-1, yielding plasmid pCDF-hemLA. Fd and FNR genes from *T. elongatus* BP-1 were also designed as chimeric genes, artificially synthesized and ligated into pETduet-1, yielding the plasmid pET-Fd-FNR. For fine turning expression of Fd-FNR, RBS was designed with online software (http://voigtlab.ucsf.edu/software, accessed on 15 May 2022) to control the translation of the protein at desired levels. Site-directed mutations of RBS were performed by reversed PCR using pET-Fd-FNR as template. The PCR product was digested by DpnI and transformed into competent *E. coli* Top 10, generating a series of plasmid mutants of pET-Fd-FNR. These pET-Fd-FNR mutants were individually co-transformed with pRSF-BHo1-BvdR into *E. coli* BL21(DE3), generating strains M1FF1, M1FF2, M1FF3, M1FF4, and M1FF5. NADPH-regenerating genes *ZWF* (coding for glucose-6-phosphate 1-dehydrogenase), *nadK* (NAD kinase), *gdhA* (NADP-specific glutamate dehydrogenase), and *IDH* (isocitrate dehydrogenase) were amplified from *E. coli* genomic DNA, digested with BamHI, and individually ligated into similarly restriction enzyme-digested plasmids pETDuet-1 or pACYCduet-1, yielding plasmids pET-ZWF, pET-IDH, pET-NAD, pET-gdhA, pACYC-ZWF, pACYC-IDH, pACYC-NAD, and pACYC-gdhA, respectively. The plasmids pETDuet-1, pET-ZWF, pET-IDH, pET-NAD, and pET-gdhA were individually co-transformed with pRSF-BHo1-BvdR into *E. coli* BL21(DE3), generating strains M1ET, M1ZE, M1IE, M1NE, and M1GE, respectively. The plasmids pACYCDuet-1, pACYC-ZWF, pACYC-IDH, pACYC-NAD, and pACYC-gdhA were individually co-transformed with pRSF-BHo1-BvdR into *E. coli* BL21(DE3), generating strains M1AC, M1ZA, M1IA, M1NA, and M1GA, respectively. Strain M1GF was generated by co-transformation of the plasmids pRSF-BHo1-BvdR, pET-Fd-FNR, and pACYC-gdhA into *E. coli* BL21(DE3). Strain M1BHG was generated by co-transformation of the plasmids pRSF-BHo1-BvdR, pCDF-hemBH, and pACYC-gdhA into *E. coli* BL21(DE3). Strain M1BHF was generated by co-transformation of the plasmids pRSF-BHo1-BvdR, pCDF-hemBH, and pET-Fd/FNR into *E. coli* BL21(DE3).

### 4.3. Media and Culture Conditions

LB medium (10 g/L tryptone, 5 g/L yeast extract, 10 g/L NaCl) was used for *E. coli* cultivation for the DNA manipulations and seed cultures. TB medium (12 g/L tryptone, 24 g/L yeast extract, 4 g/L glycerol, 2.31 g/L KH_2_PO_4_, 12.54 g/L K_2_HPO_4_) was used for *E. coli* cultivation for BR production. Appropriate antibiotics were added to the culture medium to provide selective pressure. Single colonies of recombinant *E. coli* were inoculated in LB medium. 2 mL of overnight seed cultures were then inoculated into 40 mL of TB medium and cultured at 37 °C until the optical density at 600 nm reached 0.8. The cultures were then cooled to 30 °C, and the expression of the foreign genes was induced by the addition of isopropyl-β-D-thiogalactoside (IPTG) with a final concentration of 0.8 mM. After a further incubation for 48 h at 30 °C in the dark, the E. coli cells were harvested by centrifugation at 6000× *g* for 10 min.

Fed-batch fermentation was performed in a 5 L bioreactor (BioF6005S-G, Shanghai, China). 2.0% inoculation of overnight seed culture was transferred into the bioreactor containing 2.5 L TB medium. A constant feeding mode was employed to achieve high cell density. The feeding medium was composed of 200 g/L glucose, 5 g/L NaCl, 5 g/L MgSO_4_·7H_2_O, 7 g/L KH_2_PO_4_, and 8 g/L K_2_HPO_4_. The culture was cooled to 30 °C when OD_600_ reached 10.0. IPTG was added to the culture media with a final concentration of 0.8 mM.

### 4.4. SDS-PAGE Analysis

Expression of HO1 and BvdR in the recombinant M2 *E. coli* cells was analyzed by SDS-PAGE. The *E. coli* cells were disrupted by sonication and then centrifuged at 12,000× *g* for 10 min at 4 °C. The supernatant was mixed with loading buffer and boiled for 10 min. Ten microliters of the samples was loaded on 15% acrylamide gel. Resolved proteins were visualized by staining with Coomassie Blue.

### 4.5. HPLC and LC–MS Analysis of BR

*E. coli* cells were washed twice with distilled water and extracted by methanol in darkness for two hours. The extractions were filtered through a 0.22 μm polytetrafluoroethylene syringe filter and then resolved by reversed-phase chromatography using an Agilent Technologies 1260 system. The HPLC column was a 4.6 × 250 mm Phenomenex Ultracarb (Torrance, CA, USA). The mobile phase consisted of acetonitrile and water (containing 0.1% trifluoroacetic acid) in a 95:5 ratio (*v*/*v*), at a rate of 0.8 mL/min. The column temperature was set at 30 °C. The absorbance was measured at 450 nm. Peak areas were quantified using Agilent Openlab CDS software (Ver 2.6.0.691). For further analysis, LC–MS was performed by using an LCQ FLEET device (Thermo Scientific, Waltham, MA, USA). Detection conditions were flow rate of 0.6 mL min^−1^ and column temperature of 25 °C.

### 4.6. Statistical Analysis

The fermentation experiments were carried out in triplicate, and data were calculated to obtain the mean value and standard deviation. Statistical analyses were performed using SPSS 17.0 software (SPSS Inc., Chicago, IL, USA). The difference was evaluated using an independent *t* test. Differences were considered significant at a probability level of *p* < 0.05.

## Data Availability

Data will be available upon request.

## Supplementary Materials

The following supporting information can be downloaded at: https://www.mdpi.com/article/10.3390/ijms25179741/s1.
