# Supplementary material for: Metabolic Engineering of Escherichia coli for Production of a Bioactive Metabolite of Bilirubin
Source: Int J Mol Sci. 2024 Sep 9;25(17):9741. doi: 10.3390/ijms25179741 (PMC11396004; doi:10.3390/ijms25179741)
Supplement: Supplementary file 1 [file ijms-25-09741-s001.zip › ijms-3159679-supplementary.pdf]

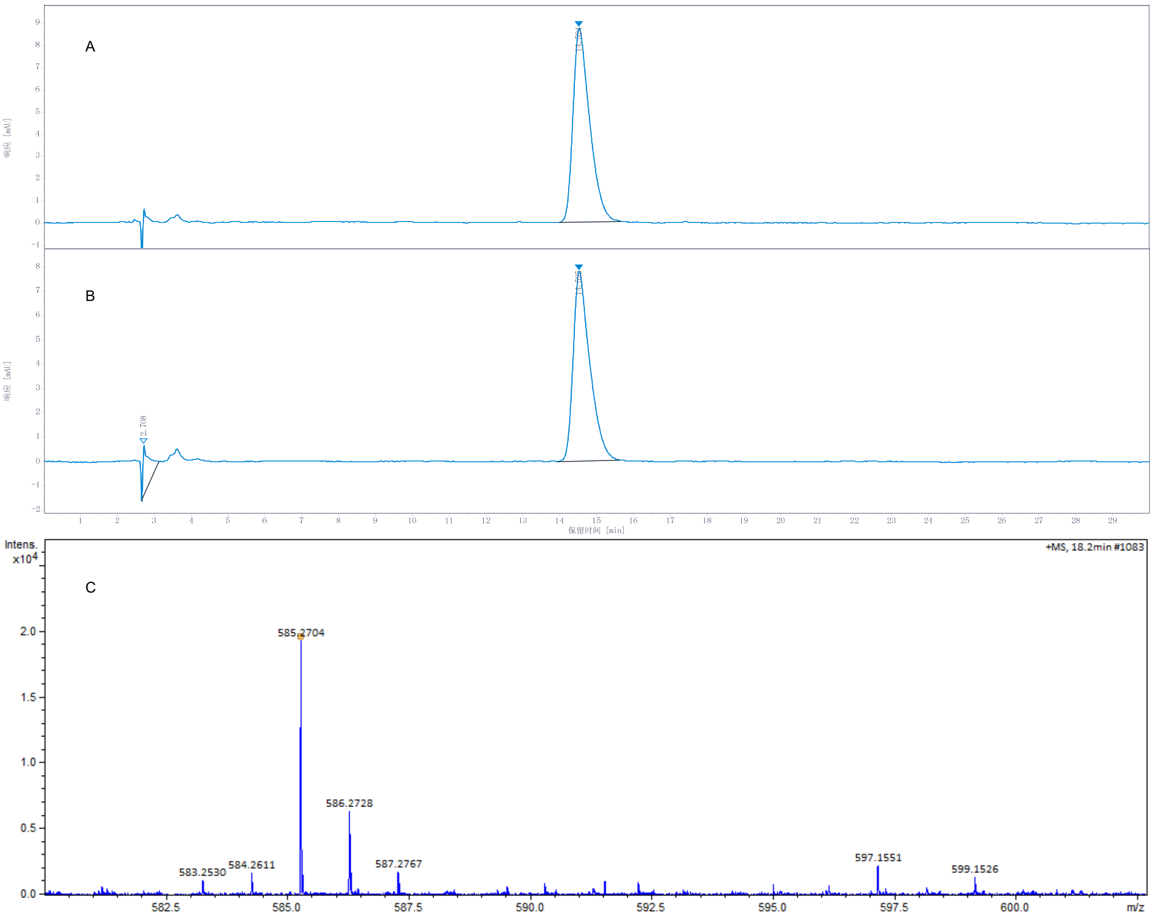

Figure S1. HPLC chromatograms (A, B) and LC-MS spectrum (C) of methanol extracts from recombinant *E. coli* cells. A: stand bilirubin dissolved in methanol. B: methanol extract from strain M2 cells. C: LC-MS spectrum of chromatograms of methanol extract from strain M2 cells.

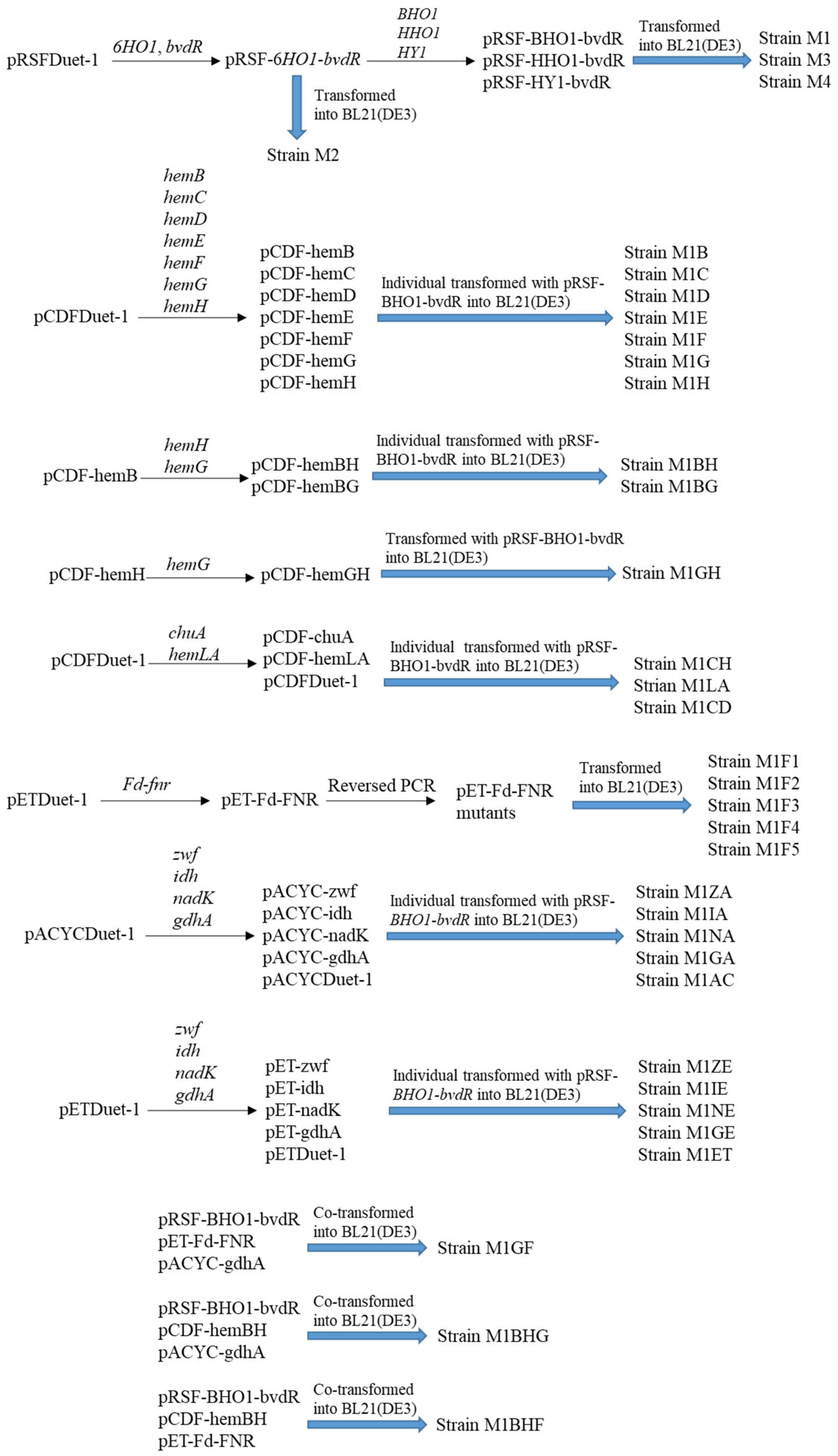

Figure S2 Flow diagram of the construction of plasmids and strains.

Table S1 Recombinant *E. coli* strains and plasmids used in this study.

Strains	Description
M1	*E. coli* BL21(DE3) harboring pRSF-BHo1-BvdR
M2	*E. coli* BL21(DE3) harboring pRSF-6Ho1-BvdR
M3	*E. coli* BL21(DE3) harboring pRSF-HBHo1-BvdR
M4	*E. coli* BL21(DE3) harboring pRSF-YBHo1-BvdR
E1	*E. coli* BL21(DE3) harboring pRSFDuet-1
M1CD	*E. coli* BL21(DE3) harboring pRSF-BHo1-BvdR and pCDFDuet-1
M1CH	*E. coli* BL21(DE3) harboring pRSF-BHo1-BvdR and pCDF-chuA
M1LA	*E. coli* BL21(DE3) harboring pRSF-BHo1-BvdR and pCDF-hemLA
M1B	*E. coli* BL21(DE3) harboring pRSF-BHo1-BvdR and pCDF-hemB
M1C	*E. coli* BL21(DE3) harboring pRSF-BHo1-BvdR and pCDF-hemC
M1D	*E. coli* BL21(DE3) harboring pRSF-BHo1-BvdR and pCDF-hemD
M1E	*E. coli* BL21(DE3) harboring pRSF-BHo1-BvdR and pCDF-hemE
M1F	*E. coli* BL21(DE3) harboring pRSF-BHo1-BvdR and pCDF-hemF
M1G	*E. coli* BL21(DE3) harboring pRSF-BHo1-BvdR and pCDF-hemG
M1H	*E. coli* BL21(DE3) harboring pRSF-BHo1-BvdR and pCDF-hemH
M1BH	*E. coli* BL21(DE3) harboring pRSF-BHo1-BvdR and pCDF-hemBH
M1BG	*E. coli* BL21(DE3) harboring pRSF-BHo1-BvdR and pCDF-hemBG
M1GH	*E. coli* BL21(DE3) harboring pRSF-BHo1-BvdR and pCDF-hemGH
M1ET	*E. coli* BL21(DE3) harboring pRSF-BHo1-BvdR and pETduet-1
M1NE	*E. coli* BL21(DE3) harboring pRSF-BHo1-BvdR and pET-NAD
M1IE	*E. coli* BL21(DE3) harboring pRSF-BHo1-BvdR and pET-IDH
M1GE	*E. coli* BL21(DE3) harboring pRSF-BHo1-BvdR and pET-gdhA
M1ZE	*E. coli* BL21(DE3) harboring pRSF-BHo1-BvdR and pET-ZWF
M1AC	*E. coli* BL21(DE3) harboring pRSF-BHo1-BvdR and pACYCduet-1
M1NA	*E. coli* BL21(DE3) harboring pRSF-BHo1-BvdR and pACYC-NAD
M1IA	*E. coli* BL21(DE3) harboring pRSF-BHo1-BvdR and pACYC-IDH
M1GA	*E. coli* BL21(DE3) harboring pRSF-BHo1-BvdR and pACYC-gdhA
M1ZA	*E. coli* BL21(DE3) harboring pRSF-BHo1-BvdR and pACYC-ZWF
M1FF1	*E. coli* BL21(DE3) harboring pRSF-BHo1-BvdR and pET-RBS8-Fd/FNR
M1FF2	*E. coli* BL21(DE3) harboring pRSF-BHo1-BvdR and pET-RBS75-Fd/FNR
M1FF3	*E. coli* BL21(DE3) harboring pRSF-BHo1-BvdR and pET-RBS769-Fd/FNR
M1FF4	*E. coli* BL21(DE3) harboring pRSF-BHo1-BvdR and pET-RBS3369-Fd/FNR
M1FF5	*E. coli* BL21(DE3) harboring pRSF-BHo1-BvdR and pET-RBS4043-Fd/FNR
M1GF	*E. coli* BL21(DE3) harboring pRSF-BHo1-BvdR, pET-Fd/FNR and pACYC-gdhA
M1BHG	*E. coli* BL21(DE3) harboring pRSF-BHo1-BvdR, pCDF-hemBH, pACYC-gdhA
M1BHF	*E. coli* BL21(DE3) harboring pRSF-BHo1-BvdR, pCDF-hemBH, pET-Fd/FNR
